# Potentially Inappropriate Prescribing to Older Patients Admitted to Units for Integrated Continuous Care: Application of STOPP/START Criteria

**DOI:** 10.3390/jcm14092861

**Published:** 2025-04-22

**Authors:** Catarina Candeias, Jorge Gama, Márcio Rodrigues, Sara Meirinho, Amílcar Falcão, Miguel Castelo-Branco, Gilberto Alves

**Affiliations:** 1 RISE-Health, Department of Medical Sciences, Faculty of Health Sciences, University of Beira Interior, Av. Infante D. Henrique, 6200-506 Covilhã, Portugal; catarinacandeias4@gmail.com (C.C.); sara.meirinho@ubi.pt (S.M.); mcbranco@fcsaude.ubi.pt (M.C.-B.); 2 ULSCB—Castelo Branco Local Health Unit, Av. Pedro Álvares Cabral, 6000-084 Castelo Branco, Portugal; 3 CMA-UBI—Centre of Mathematics and Applications, Faculty of Sciences, University of Beira Interior, Rua Marquês D’Ávila e Bolama, 6201-001 Covilhã, Portugal; jgama@ubi.pt; 4 BRIDGES—Biotechnology Research, Innovation and Design for Health Products, Polytechnic University of Guarda, Avenida Dr. Francisco Sá Carneiro, n.º 50, 6300-559 Guarda, Portugal; 5 CIBIT—Coimbra Institute for Biomedical Imaging and Translational Research, University of Coimbra, Pólo das Ciências da Saúde, Azinhaga de Santa Comba, 3000-548 Coimbra, Portugal; acfalcao@ff.uc.pt; 6 Laboratory of Pharmacology, Faculty of Pharmacy, University of Coimbra, Pólo das Ciências da Saúde, Azinhaga de Santa Comba, 3000-548 Coimbra, Portugal; 7 CACB—Academic Clinical Center of Beiras, Faculty of Health Sciences, University of Beira Interior, Av. Infante D. Henrique, 6200-506 Covilhã, Portugal; 8 ESALD-IPCB—Dr. Lopes Dias School of Health, Polytechnic Institute of Castelo Branco, Av. do Empresário, Campus da Talagueira, 6000-767 Castelo Branco, Portugal; 9 UFBI—Pharmacovigilance Unit of Beira Interior, University of Beira Interior, Av. Infante D. Henrique, 6200-506 Covilhã, Portugal

**Keywords:** potentially inappropriate medications, potential prescribing omissions, STOPP/START criteria, aged 75 and over, aged 85 and over, Portuguese elderly patients

## Abstract

**Background**: Potentially inappropriate medications (PIMs) and potential prescription omissions (PPOs) have been widely explored, but few studies focused on patients aged 75 years and over. This study was planned to explore the demographic and clinical characteristics of the older patients admitted to Units for Integrated Continuous Care, and to assess the prevalence and potential predictors of PIMs and PPOs. **Methods**: An observational, retrospective, and multicenter study was performed on 135 patients aged 75 years or older (i.e., 75–84 years and ≥85 years). PIMs and PPOs were investigated by applying the Screening Tool of Older People’s Prescriptions (STOPP) and Screening Tool to Alert to Right Treatment (START) criteria. **Results**: The oldest-old patients (≥85 years) were less likely to come from a hospital, had fewer daily medications and a lower number of oral doses, but they presented a higher Charlson Comorbidity Index, were more dependent on activities of daily living, and were less obese than those aged 75–84 years. Results showed a high prevalence of PIMs and PPOs in both age groups. The more common PIMs and PPOs were the same in both age groups. The oldest-old patients who suffered falls were more likely to have a prescription omission of vitamin D supplements. The PIM index was not significantly different between age groups but was higher in the oldest-old group. **Conclusions**: Patients with a higher number of prescriptions had a higher risk of PIMs. Regarding PPOs, male gender and fall risk were predictors in the youngest group, while the number of comorbidities was significantly associated with PPOs in the oldest group. This study supports the usefulness of the STOPP/START criteria to identify PIMs and PPOs in these patients, but more research is required to determine the potential adverse outcomes of PIMs and PPOs and their clinical and economic consequences.

## 1. Introduction

Optimization of drug therapy in the elderly population is a well-known goal that can be accomplished through the prescription of appropriate drugs. In contrast, potentially inappropriate drugs prescribed to older people that have no clear indication based on evidence can lead to higher risks of developing adverse effects [1]. Considering the physiological characteristics and the existence of chronic conditions that are common in the oldest patients, the side effects of inappropriate prescribed drugs can be serious in this population. There are still a higher number of drugs prescribed to older people that can be problematic, such as anticholinergic, benzodiazepines, non-steroidal anti-inflammatory drugs, antipsychotics, and antibiotics [2]. In fact, the clinical and economic impact of mis/over/underuse of drugs is not only a current issue, but also a future problem that increases with the aging of the population. Worldwide, life expectancy at birth reached 73.3 years in 2024, reflecting an increase of 8.4 years since 1995 [3]. By 2030, one in six people worldwide will be aged 60 or over. During this period, the number of people aged 60 and over will increase from 1 billion in 2020 to 1.4 billion. By 2050, the global population of people aged 60 and over is expected to double to 2.1 billion. In addition, the number of people aged 80 and over is expected to triple between 2020 and 2050, reaching 426 million [4].

The increase in population aging is usually followed by an increase in comorbidities, consequently leading to the use of more medications. A retrospective cross-sectional study conducted by Mo et al. [5] revealed that patients aged 80 years or older had statistically significant higher comorbidities, medical prescription, hospitalization expenses, length of stay, and mortality than those aged 65–79 years old. In addition to this, the same study also showed that the group of the oldest patients had more prescriptions of benzodiazepines, anticholinergics, megestrol, antipsychotics, theophylline, and aspirin. Therefore, it is essential to explore the use of potentially inappropriate medications (PIMs), particularly in older age groups.

Several tools have been developed, published, and updated in the last years to study the inappropriate use of medication in older patients. One of these tools are the explicit criteria developed by Beers et al. [6] and their updates [7,8,9,10,11]. However, as almost half of the drugs included in the Beers criteria are not commercialized in most European countries, their clinical applicability has been questioned [12]. Instead, the reviewed Screening Tool of Older Persons’ Prescriptions and Screening Tool to Alert Doctors to Right Treatment (STOPP/START) criteria [13] demonstrated to cover significantly more PIMs that contribute to hospital admissions, also enabling the detection of PIMs and the identification of potential prescription omissions (PPOs) that may also have harmful clinical consequences [14,15]. Furthermore, it was also reported that the 2015 update (STOPP/START version 2) identified more PIMs of clinical relevance [16], and, more recently, version 3 [17] has shown improvements [18,19].

As polypharmacy and comorbidities have been widely reported as predictors of PIMs [20,21,22,23,24,25,26] and PPOs [21,25,27,28], respectively, a higher prevalence of PIMs and PPOs is expected to be found in older patients. Mostly due to their frailer healthcare status, these patients should be more supervised by healthcare professionals, which may contribute to avoiding inappropriate prescriptions. Nevertheless, care must be taken at the time of applying different criteria to identify possible PIMs and PPOs. This is particularly true for the oldest and frailest patients, as both the validity and reliability of the criteria in this population can be somewhat controversial. An example of this is that contrary to STOPP criteria where the application to patients aged ≥ 65 years seems to be the only requirement, the START criteria safeguard patients at the end of life [13].

Considering the elderly population, with specific or diverse comorbidities, and living or not in an institutional setting, the literature is already extensive regarding these issues. However, most studies have included all patients in one age group without exploring potential differences in clinical characteristics and drug use among elderly people of different ages [24,25,29,30,31,32,33]. Besides that, few studies restricted a minimum age cut-off above 75 years old [14,34,35,36], or compared different age groups of the same population in order to establish conclusions about the oldest patients [36]. Nevertheless, after applying the STOPP/START criteria, Liu et al. [37] found that an age above 75 years old increased the risk of PIM, particularly for those aged ≥ 85 years. In addition, other studies have also found a significant association between PPOs and older age [31,38].

With this in mind, the primary objective of this study was to determine and compare the prevalence of PIMs and PPOs (overall and per individual STOPP and START criteria, respectively) among the patients aged between 75 and 84 years old, and those aged 85 years or older from Units for Integrated Continuous Care (*Unidades de Cuidados Continuados*, UCCIs). The secondary objective was to identify potential predictors of PIMs and PPOs among the demographic and clinical features of elderly patients of both age groups hospitalized in UCCIs.

## 2. Materials and Methods

### 2.1. Study Design, Setting, and Participants

An observational, retrospective, multicenter study was conducted on patients hospitalized in UCCIs included in the Portuguese National Network for Long-term Integrated Care (*Rede Nacional de Cuidados Continuados Integrados*, RNCCI). Patients were randomly selected from eight UCCIs that provided post-acute and long-term care and were discharged between June 2015 and April 2016. The protocol of this study was approved by the Ethics Committee of the Faculty of Health Sciences of the University of Beira Interior (CE-FCS-2015-030). The retrospective nature of this study did not affect any healthcare aspect of the inpatients, whereby informed consent was not required. Patient data were anonymized by attributing an alphanumeric code, and data access was restricted to the first author, who was responsible for the application of STOPP/START criteria. Statistical analysis was performed exclusively with the coding used.

### 2.2. Data Sources

Patients’ clinical information was accessed through an online software where data were recorded: periodic evaluations registered by doctors, nurses, and other health professionals of the multidisciplinary team, providing information on patients’ clinical status, diagnosis, and prescriptions. Diagnoses were collected through ICD-9-CM (International Classification of Disease, Ninth Revision, Clinical Modification) [39] and others only using the information provided in the online clinical process. The therapeutic list was initially extracted from each patient’s discharge letter or from clinical evaluations, being then coded according to the Anatomical Therapeutic Chemical (ATC) classification system. No patient was excluded due to missing data: whenever information was not available in the selected primary source, other sources were consulted to complete missing or incomplete online information.

### 2.3. Data Collection and Analysis

Demographic characteristics (age, gender, origin, length of institution stay, and discharge) and clinical features [enteral nutrition, chronic medications, daily doses, chronic conditions, Charlson Comorbidity Index (CCI) [40], and geriatric syndromes] of patients aged 74–85 years (the youngest group) and over 85 years (the oldest group) were compared. Among geriatric syndromes, polypharmacy, number of comorbid diseases ≥ 2, CCI ≥ 6, dependency in activities of daily life (ADL) assessed using the Barthel index, risk of falls, malnutrition/anorexia, obesity, pressure ulcers, and history of recent fractures were included.

Comorbid diseases included only those considered in the CCI (myocardial infarction, congestive heart failure, peripheral vascular disease, cerebrovascular disease, dementia, chronic obstructive pulmonary disease, connective tissue disease, peptic ulcer disease, mild liver disease, diabetes, hemiplegia, moderate or severe renal disease, diabetes with end-organ damage, any non-metastatic solid tumor, leukemia, malignant lymphoma, moderate or severe liver disease, metastatic solid tumor, and AIDS). However, to determine the most common chronic conditions, other diseases were also analyzed: angina, arrhythmia, valvular disease, hypertension, Parkinson’s disease, renal disease either severe or not, urinary incontinence, recurrent urinary tract infection, rheumatoid arthritis, rheumatic disease, osteoporosis, depression, constipation, benign prostatic hypertrophy, and glaucoma. The prevalence of chronic conditions and prescribed therapeutics in each subgroup was analyzed and calculated as a proportion of all patients and of each studied age group (75–84 years old and ≥85 years old).

Despite the different meanings associated with the term polypharmacy, in this study, polypharmacy was defined as the chronic use of 5 or more medications per day [41]. The CCI [40], a morbidity index that considers the number of comorbid conditions and their severity, was also calculated to investigate comorbidities in each studied group.

The prevalence of PIMs and PPOs were found through the application of the overall STOPP and START criteria, respectively, that were explored and compared in both age groups. For that, continuous variables (the number of PIMs and PPOs identified) and categorical variables (with or without PIM and/or PPO) were considered. Also, the PIM index, which is calculated by dividing the number of PIMs by the number of prescribed medications, is another tool that can complement the analysis of PIM prevalence [42].

Demographic characteristics, clinical features (including all geriatric syndromes), and chronic conditions were also analyzed as potential predictors of PIMs and PPOs in patients aged 75–84 years and those aged ≥ 85 years.

In descriptive statistics, continuous variables were expressed as mean ± standard deviation, as median, and as the interquartile range (P25; P75). On the other hand, categorical variables were expressed as the number of observations (absolute frequency) and percentages (relative frequency).

The Kolmogorov–Smirnov test was used to determine whether continuous variables had a normal distribution, and Student’s *t*-test or the Mann–Whitney test was used depending on the result. In contrast, variables were compared using Chi-Square or Fisher’s Exact Tests. The existence of associations between PIMs or PPOs and the other variables was first tested with a bivariate logistic regression and the respective ORs were estimated. Then, to identify the determinants of PIMs and PPOs, variables with a significant association with PIMs or PPOs were tested in a multivariate analysis. ORs were adjusted for sex and for the significant variables previously found and the logistic regression analysis, with logit link function, was performed using the forward selection method based on the likelihood ratio (significance level of 5% for a variable entered and 10% for its removal) to find independent predictors associated with PIMs or PPOs. The Hosmer–Lemeshow test was performed to assess the goodness of fit, whereas the ROC curve allowed the evaluation of the model’s discriminatory power and its sensitivity/specificity. Differences were considered statistically significant when *p* < 0.05 and the confidence interval (CI) was 95%. Statistical analyses were performed using IBM SPSS Statistics version 27.

## 3. Results

### 3.1. Characteristics of the Study Population

The demographic characteristics and clinical features of patients are detailed in Table 1. Of the 135 patients studied, 79 were aged between 75 and 84 years old (median age of 80 [78; 82] years old), while 56 were aged ≥ 85 years old (median age of 89 [86; 91] years old). Among those aged between 75 and 84 years old, 68.4% were female, most came from the hospital (58.2%), had a median length of stay of 90 [45; 146] days, and most returned to their residence (48.1%). However, a considerable number of patients died in the UCCI or soon after emergency hospital admission (20.3%). Patients aged ≥ 85 years old were also predominantly female (66.1%), arising from either a residence (46.4%) or a hospital (41.1%), had a length of stay median of 102 [89; 173] days, and also had a higher discharge to residence (28.6%). The youngest age group was more likely to come from the hospital (*p* = 0.018).

Considering the prevalence of patients fed through enteral nutrition and also the prevalence of comorbid diseases, no significant differences were found between patients aged 75–84 years old and ≥85 years old. Instead, patients aged ≥ 85 years old had fewer chronic medications prescribed (*p* = 0.029), fewer doses per day (*p* = 0.020), and higher CCI (*p* = 0.046). Concerning geriatric syndromes, patients aged ≥ 85 years old were more likely to have a CCI ≥ 6 (*p* = 0.027), were more dependent on ADL (*p* = 0.001), and were less obese (*p* = 0.001). No significant differences were found for the following variables: polypharmacy, two or more comorbid diseases, fall risk (medium or high), malnutrition/anorexia, the existence of pressure ulcers, or a history of recent fractures.

Table 2 presents all the information regarding the most common chronic conditions and most prescribed therapeutic subgroups in the studied population. The most common chronic conditions in patients aged 75–84 years old were hypertension (67.1%), diabetes mellitus (40.5%), depression (38.0%), and constipation (35.4%). At the same time, the most commonly prescribed drugs were antithrombotic agents (77.2%), drugs for acid-related disorders (68.4%), psycholeptics (65.8%), and psychoanaleptics (64.6%). In patients aged ≥ 85 years old, hypertension (71.4%), urinary incontinence (42.9%), cerebrovascular disease (33.9%), and dementia (33.9%) were the most common chronic conditions. In this age group, the most commonly prescribed drugs were drugs for acid disorders (69.6%), psycholeptics (67.9), antithrombotic agents (62.5%), and diuretics (55.4%). The youngest patients were more likely to have diabetes mellitus (*p* = 0.020) and depression (*p* = 0.041), and to take psychoanaleptics (*p* = 0.007), drugs for diabetes treatment (*p* = 0.012), and antiepileptics (*p* = 0.002). Older patients were more likely to have urinary incontinence (*p* = 0.008).

### 3.2. Potentially Inappropriate Medications

Table 3 shows the prevalence of PIM and PPO in both age groups. In patients aged 75–84 years old, the median number of PIMs was 3 [1; 4], and the prevalence of PIM was 88.6%. In patients aged ≥ 85 years old, a median of 2 [1; 3.75] PIMs were identified per patient, and the prevalence of PIM was 85.7%. Almost three-quarters of the patients in both age groups had two or more PIMs, but no significant difference was found between patients aged 75–84 years old and those aged ≥ 85 years old. The PIM index was 0.273 [0.143; 0.429] for the youngest elderly and 0.333 [0.157; 0.441] for the oldest, with no statistically significant difference.

The most common PIMs in both age groups (with their prevalence in patients aged 75–84 years old vs. ≥85 years old, respectively) were the following: benzodiazepines as drugs that predictably increase the risk of falls (54.4% vs. 51.8%); benzodiazepines for longer than four weeks (50.6% vs. 50.0%) and neuroleptics in elderly prone to falls (25.3% vs. 23.2%) (Table 4). However, no statistically significant differences were found among these PIMs.

In the multivariate logistic regression, a statistically significant association was found between the number of medications [OR = 1.71, 95%CI: 1.19–2.48), *p* = 0.004] and the diagnosis of cerebrovascular disease [OR = 0.16 (95% CI 0.03–0.91), *p* = 0.038] in the group aged 75–84 years old. There was also a statistically significant association with the number of medications [OR = 1.44 (95% CI 1.04–2.01), *p* = 0.028] and the diagnosis of chronic pulmonary disease [OR = 0.12 (95% CI 0.02–0.90), *p* = 0.039] for those aged ≥ 85 years old. Logistic regression information is presented in Table 5 (univariate level) and Table 6 (multivariate level).

**Figure 1:**
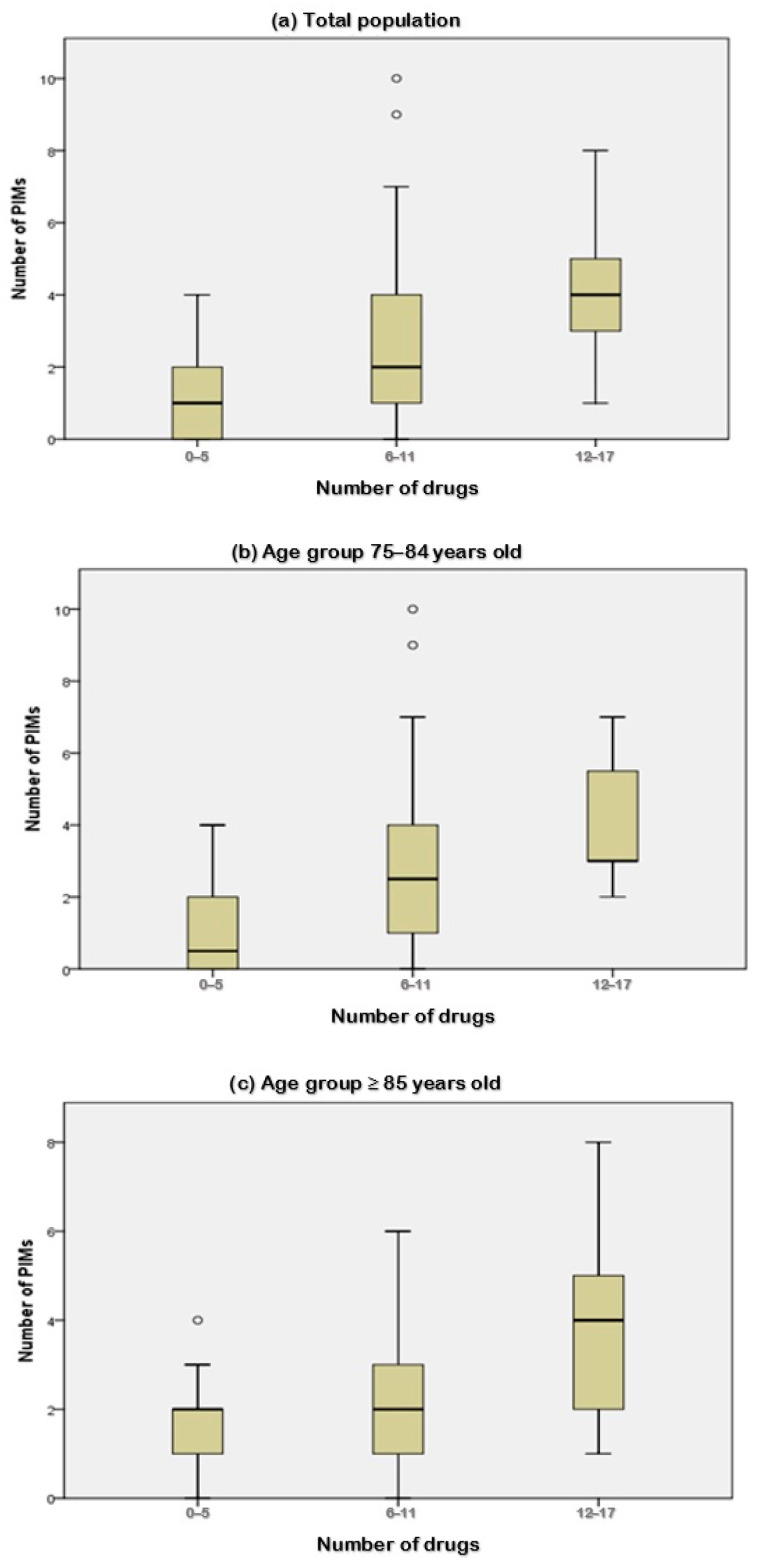
Relationship between the number of potentially inappropriate medications (PIMs) and the number of drugs, according to age groups. Box-and-whisker plots showing the relationship between the number of PIMs and the number of drugs prescribed in (**a**) all population, (**b**) patients aged 75–84 years old, and (**c**) patients aged ≥ 85 years old.

As the number of drugs prescribed was the only predictor of PIM for both age groups, the relationship between this variable and the number of PIMs was evaluated. A statistically significant association was found for the whole population (*p* = 0.020), for patients aged 75–84 years old (*p* = 0.001), and for patients aged ≥ 85 years old (*p* = 0.018). Figure 1 shows the corresponding box-and-whisker plots, and the results of multiple comparisons performed using the Kruskal–Wallis test.

### 3.3. Potential Prescribing Omissions

A median of 2 [1; 3] PPOs was found in patients aged between 75 and 84 years old. Almost half had more than one PPO, and the prevalence of PPO was 79.7%. The oldest old had a median of 2 [1; 3] PPOs, almost 75% had at least 2 PPOs, and the prevalence of PPO was 85.7%. No significant differences were found between both age groups (Table 3).

The most common PPOs and their respective prevalence among patients aged between 75 and 84 years old and among patients with ≥85 years old were the following: vitamin D supplements in the oldest that are housebound, experiencing falls, or with osteopenia (35.4% vs. 55.4%); vitamin D–calcium supplement in patients with osteoporosis and/or previous fragility fracture(s) (24.1% vs. 33.9%); and angiotensin-converting enzyme inhibitor with systolic heart failure and/or documented coronary artery disease (17.7% vs. 21.4%) (Table 4). The oldest age group with a history of falls showed higher potential omission of vitamin D supplements (*p* = 0.022) than those aged between 75–84 years old.

In the age group between 75 and 84 years old, a statistically significant association was found between the male gender [OR = 14.41 (95% CI 1.55–134.47), *p* = 0.019] and the fall risk [OR = 5.72 (95% CI 1.21–27.05), *p* = 0.028]. However, in the oldest group, only the number of comorbidities [OR = 2.50 (95% CI 1.02–6.14), *p* = 0.046] demonstrated to be a predictor of PPO (Table 6).

## 4. Discussion

### 4.1. Main Findings

Patients aged ≥ 85 years old were less likely to come from a hospital, had fewer daily medications, and had fewer oral doses, but had a higher CCI, were more likely to have a CCI ≥ 6, were more dependent on ADL, and were less obese than those aged between 75 and 84 years old.

Regarding comorbidities, hypertension was the most common chronic condition present in both age groups. In patients aged 75–84 years old, diabetes mellitus, depression, and constipation followed hypertension as existent comorbidities. In the oldest group, the other conditions that were more prevalent were urinary incontinence, cerebrovascular disease, and dementia. Antithrombotic agents, drugs for gastric acid-related disorders, and psycholeptics were the therapeutic subgroups more commonly prescribed in both age groups, although with a distinct hierarchy. Drugs used for the treatment of hypertension were not in the top three, probably because of the different clinical approaches that can be chosen (e.g., diuretics, agents acting on the renin–angiotensin system, beta-blocking agents, and cardiac insufficiency therapy). Psycholeptics had a high prevalence in both age groups, even though they are a therapeutic subgroup directly related to PIM in the elderly, once they predictably increased the risk of falls. In addition, drugs for gastric acid-related disorders are also commonly prescribed, despite fewer patients having a history of peptic ulcer. However, this may be related to the use of drugs that can be harmful to the gastrointestinal system, such as antithrombotic agents, corticosteroids, and non-steroidal anti-inflammatory drugs (NSAIDs).

PIM and PPO were ubiquitous in the youngest studied group (88.6% and 79.7%, respectively) and also in the oldest group (85.7% for both). Still, no statistically significant differences were found. This may be related to the additional concern that most physicians may have at the time of prescribing PIM drugs to older patients, which may lower the PIM prevalence in the oldest studied population. Nevertheless, the PIM index was higher among the oldest, which may mean that patients aged 75–84 years old may have received more appropriate prescriptions [42]. Concerning PPO, there were also no statistically significant differences in the youngest group, in contrast to the higher prevalence of PPO found in the oldest group, which may be related to the higher CCI.

The most common PIMs were the same in both age groups (benzodiazepines as drugs that predictably increase the risk of falls, benzodiazepines for ≥ 4 weeks, and neuroleptics as drugs that predictably increase the risk of falls). The same trend was observed for PPOs between the two age groups studied here (vitamin D supplement in older people who are housebound or experiencing falls or with osteopenia; vitamin D and calcium supplement in patients with known osteoporosis and/or previous fragility fracture(s), and angiotensin-converting enzyme inhibitor with systolic heart failure and/or documented coronary artery disease). Thus, assuming that an intervention was carried out that resulted in the elimination of the three most common PIMs and PPOs, it is possible that this reevaluation would reduce their prevalence of PIMs and PPOs in the youngest group up to 74.7% and 62.0%, respectively, and in the oldest group up to 64.3% in both cases. In this way, PIMs and PPOs would be absent in 13.9% of patients aged between 75 and 84 years old (against 3.8%) and in 10.7% of patients aged ≥ 85 years old (against 3.6%).

The number of medications was a predictor of the existence of PIM in both age groups. At the same time, this variable was also found to be associated with prevalence of cerebrovascular disease in patients aged 75–84 years old and with the prevalence of chronic pulmonary disease in those aged ≥ 85 years old. Considering the total population studied, and both the youngest and the oldest groups, the total number of medications had a significant relationship with the number of PIMs found. For PPO, male gender and fall risk were predictors in the youngest group, whereas the number of comorbidities was significantly associated with PPO in the oldest group.

### 4.2. Comparing with Existing Literature

National data are scarce and do not compare different age groups. However, the available data show a high prevalence of PIM values (75.4% in nursing homes [29], 74.0% in hospitalized patients [43]), and of PPO values (68.1% in a stroke unit [44]). However, from an international perspective, the reported prevalence of medication misuse, overuse, and underuse is very different for both PIMs and PPOs, ranging from 15% to 81% for PIMs [15,20,21,22,23,24,25,27,28,33,35,37,45,46,47,48,49,50,51,52] and from 23% to 74% for PPOs [21,22,24,25,27,28,35,37,38,48,49,51,52]. This diversity is probably not only the result of different healthcare practices, but also to different types of target population (oldest or frailest) in different situations (admission or discharge, with acute or chronic conditions) and in different healthcare settings (primary care, nursing home, hospital).

Regarding the comparison of studies that grouped by age, San-José et al. [36] also compared patients aged 75–84 years old with patients aged ≥ 85 years old who were admitted to the hospital. In this study, the obtained prevalence of PIM and PPO was, respectively, of 60.5% and 49.6% in the youngest group, against 63.4% and 53.7% in the oldest group. Although these are the lowest results, they could be much closer to our results by reassessing the six criteria (three PIMs and three PPOs most prevalent) mentioned above. In fact, this study is undoubtedly one of the studies focusing on the oldest patients with the highest prevalence of PIM and PPO. In the elderly aged ≥ 80 years old, Dalleur et al. [34] found a prevalence of 59% for PIM and 41% for PPO, and Wauters et al. [14] revealed misuse of 67% and underuse of 56%. They also pointed out that PIMs and PPOs coexisted in 40% of the cases and were absent in 17% of patients, a better result than that found in our study (72.2% and 3.8% for the youngest group; 75.0% and 3.6% for the oldest group). However, the lowest prevalence was found in primary care patients, who had lower levels of polypharmacy (61% and 58%, respectively) compared to both age groups of inpatients included in San-José et al. [36] (90.6% and 93.4%, respectively) and our study (92.4% and 89.3%, respectively).

Intriguingly, some studies of the oldest elderly presented a higher prevalence of PIM than PPO [14,34,36]. This contrasts with the results of our study, which found a higher prevalence of PPOs in the youngest group compared with the oldest group, where PPOs had a prevalence 6% greater than PIMs. Moreover, the only statistical difference found between the age groups was in the most common PPO (vitamin D supplement in older people who are housebound or experiencing falls or with osteopenia), showing that the oldest group was more likely to have a prescription omission of vitamin D supplement than the youngest group. These findings may be due to a greater reluctance to add prescriptions for older patients who are already heavily medicated.

Regarding the most common PIMs and PPOs, the results obtained were not surprising. In fact, the literature often mentions benzodiazepines and neuroleptics as the chemical subgroups involved in the most common PIMs [20,23,24,35,36,37,46,50,51,52], as well as vitamin D supplements, combined or not with calcium, as the drugs that more contribute to PPOs [28,35,36,38,51,52,53].

In the literature, polypharmacy may be one of the most frequently mentioned factors as a predictor of PIMs. However, the number of prescribed medications has also been identified in previous studies as a potential factor contributing to PIMs [25,27,28,49,50], being a common predictor of PIM in both age groups. Still, the negative association of cerebrovascular disease in the youngest-old and chronic pulmonary disease in the oldest-old requires further investigation.

Concerning PPO, the oldest group had the number of comorbidities as a predictor. However, the comparison with the literature is more difficult to establish as the authors tested different variables related to different comorbidities. Nevertheless, several studies found predictors of PPO associated with comorbidity, either CCI [25,27], CCI ≥ 2 [21,54], or multimorbidity [28,36]. For the youngest group, the male gender was more likely to have PPOs, which has also been reported in other studies [22], but it is not consistent in the literature [38].

### 4.3. Strengths and Limitations

The multicenter character of this study is a strength, as patients from eight different UCCIs of post-acute and long-term care were included. However, the small sample size may be the main limitation of the present study, preventing the generalization of the results obtained. Furthermore, being an observational study, it does not allow for the exploration of possible improvements that could be achieved through reviewing patients’ therapy and analyzing the potential outcomes obtained with this intervention.

Data were mainly collected using an online tool used by each UCCI, which contributed to the homogeneity of the information obtained. However, it could only be complemented when other sources were available.

The selection of a subset of STOPP and START indicators, rather than the total, was a valid strategy to avoid analyzing potentially incomplete information. Despite further difficulties in comparisons between studies, this practice has also been successfully applied in other published studies [22,23,55].

All referred diagnoses (coded by ICD-9-CM or not) were analyzed to gather all patients’ comorbidities, but a time reference was not always available. In addition, for the counting of comorbid diseases, only those included in the CCI were considered, which does not allow the achievement of the total number of conditions for each patient. This finding could justify the highest prevalence of PIM in the oldest-old, as this is probably the age group with a higher number of chronic conditions.

### 4.4. Implications for Research and/or Practice

Our study also alerts to the high prevalence of PIMs and PPOs in an older population, encouraging the periodic review of patients’ therapeutic lists, with a special focus on the most common PIM and PPO and their prevalence. When the initial holistic analysis of the patient’s health status and history is made by the physician, screening at admission would probably be an appropriate strategy. However, it is also important to recognize that the STOPP/START criteria should be used as part of an overall approach in which the physician’s judgment is paramount and can never be overridden.

Despite being a study with a small sample, this may be the only Portuguese study that focuses on the oldest age group, highlighting the need for further investigation. Generally, national data exist but are scarce [29,43,44]. The recent adaptation of the STOPP/START criteria to a Portuguese version [56,57] may be an important step towards the simplification of the application of the criteria and the enrichment of national research.

### 4.5. Future Perspectives

STOPP/START and other explicit criteria have been increasingly used but more has to be done. Thus, it is essential to explore the clinical relevance of using screening tools to detect possible inappropriate drugs, performing prospective long-term studies to assess either the decrease in mis/over and underuse of medication that can be achieved with closer supervision, but also the relationship between inappropriate prescribing and negative outcomes.

The clinical usefulness of the STOPP/START criteria has been proven. Gallagher et al. [58] showed that their use significantly improved medication appropriateness in 400 hospitalized patients. Later, O’Connor et al. [59] revealed a significant reduction in the incidence of adverse drug reactions incidence and costs related to medication in the intervention group. Furthermore, Hill-Taylor et al. [60] reviewed randomized controlled studies and concluded that the STOPP/START criteria could effectively improve the quality of prescribing and clinical, humanistic, and economic outcomes.

Therefore, it is essential to improve these criteria to guarantee that the screening is quick and reliable in the quotidian practice. First, it would be crucial to describe the ICD-9 and ATC classifications for conditions and drugs, and which criterion may be included, similarly to what was already done for the first version of the criteria [61]. This will allow not only to facilitate their application in real practice, but also to become more uniform to be used among authors. Then, computerizing the clinical processes of all patients using those codes for their diagnosis and a therapeutic list would make the screening much more straightforward. It would be ideal to create software or an online tool that would allow the addition of patient information with an automatic screening of PIMs and PPOs would be ideal. Preferably, it would offer fields for selection or completion with codes (ICD-9-CM and ATC) and clinical information requested by some criteria, such as estimated glomerular filtration rate, analytical data (serum K^+^, Na^+^, Ca^2+^), and blood pressure levels. Recent trials of STOPP/START criteria software show feasibility, but direct interaction between clinicians and trained personnel is still needed to clarify specific STOPP/START recommendations for individual multi-morbid older patients [62].

The use of STOPP/START criteria may have some limitations with the existing large administrative databases. On the one hand, it should be preceded by appropriate validation [63]. On the other hand, it would only be possible to use subsets of the criteria, as some necessary information is likely to be missing. Despite that, the opportunities for large-scale research could be numerous and of paramount importance, as it could allow the investigation of the relationship between misuse, overuse, and underuse of medication with adverse drug events (e.g., hospitalization and death) and their inherent costs. Bjerre et al. [30] have shown how this could be done, an example that must be replicated in other settings.

In summary, as future challenges, involving the pharmacist in decision making would be an important step, as in many institutions, the pharmacist does not play an active or critical role in prescribing. In addition, it would be important for professionals to have software available to support prescribing and generate important alerts that could anticipate the prescription of inappropriate drugs or alert patients to conditions that require assessment of the relevance of introducing as unprescribed drug. Finally, and most importantly, it would be possible to start by publicizing this tool and even creating multidisciplinary working groups (with clinicians and pharmacists) that could in an initial phase, analyze selected patients in real time and, if it is not possible to carry out a complete screening immediately, select, for example, patients who have predictors and/or characteristics that make them more susceptible to inappropriate medication (for example, those who are more heavily medicated, older, or have a greater number of comorbidities).

## 5. Conclusions

The results of this study highlight the overall high prevalence of PIM and PPO in patients aged 75 years and older. The most common PIMs and PPOs were the same in both age groups. Still, the oldest-old (aged ≥ 85 years old) were significantly more likely to have PPO more frequently (vitamin D supplements in older people who are housebound or experiencing falls or with osteopenia). For the youngest- and oldest-old, the number of prescribed medications predicted the existence of PIM. Predictors of PPO were male gender, fall risk for the youngest old, and the number of comorbidities for the oldest-old. Analysis of the results could help plan a strategy focusing on specific targets, such as the most common PIM/PPO and patients with more medications and comorbidities. The STOPP/START criteria are a valuable tool for use in practice, but further research is needed to explore the association between inappropriate prescribing and adverse drug events as well as their costs.

## Figures and Tables

**Table 1:** Characteristics of the patients according to age groups.

	75–84 Years (*n* = 79)	≥85 Years (*n* = 56)	*p* ^a^
**Demographic characteristics**			
Age (years)			
Mean ± SD	79.8 ± 2.8	89.4 ± 4.0	
Median (P25; P75)	80 (78; 82)	89 (86; 91)	
Gender, *n* (%)			0.780 ^1^
Male	25 (31.6)	19 (33.9)	
Female	54 (68.4)	37 (66.1)	
**Medical history**			
Provenience/Origin, *n* (%)			**0.018** ^2^
Hospital	46 (58.2)	23 (41.1)	**0.049** ^1^
Residence	33 (41.8)	26 (46.4)	0.591 ^1^
Nursing home	0 (0.0)	2 (3.6)	
Primary care	0 (0.0)	3 (5.4)	
Other	0 (0.0)	2 (3.6)	
Length of stay			0.156 ^3^
Mean ± SD	146.0 ± 191.0	141.1 ± 166.9	
Median (P25; P75)	90 (45; 146)	102 (89; 173)	
Discharge to, *n* (%)			
Residence	33 (48.1)	16 (28.6)	0.342 ^1^
Death	16 (20.3)	8 (14.3)	
Another RNCCI response	13 (16.5)	11 (19.6)	
Social option/response	8 (10.1)	11 (19.6)	
Nursing home	7 (8.9)	7 (12.5)	
Other or not referred	2 (2.5)	3 (5.4)	
**Clinical features**			
Enteral Nutrition, *n* (%)			
Yes	11 (13.9)	8 (14.3)	0.953 ^1^
No	68 (86.1)	48 (85.7)	
Medication per patient			**0.029** ^4^
Mean ± SD	9.3 ± 3.1	8.0 ± 3.3	
Median (P25; P75)	9 (7; 11)	8 (6; 10)	
Number of doses			**0.020** ^3^
Mean ± SD	10.7 ± 3.9	9.2 ± 4.0	
Median (P25; P75)	11 (8; 13)	8 (6; 12)	
Comorbid diseases (CCI)			0.400 ^3^
Mean ± SD	1.8 ± 1.2	1.6 ± 1.1	
Median (P25; P75)	2 (1; 3)	2 (1; 2)	
CCI			**0.046** ^3^
Mean ± SD	5.8 ± 1.6	6.3 ± 1.5	
Median (P25; P75)	6 (5; 7)	6 (5; 7)	
Geriatric syndromes, *n* (%)			
Polypharmacy (≥5 drugs/day)			
Yes	73 (92.4)	50 (89.3)	0.553 ^2^
No	6 (7.6)	6 (10.7)	
Comorbid diseases ≥ 2			
Yes	43 (54.4)	30 (53.6)	0.921 ^1^
No	36 (45.6)	26 (46.4)	
CCI ≥ 6			
Yes	40 (50.6)	39 (69.6)	**0.027** ^1^
No	39 (49.4)	17 (30.4)	
Dependency in ADL			
Yes	67 (84.8)	56 (100.0)	**0.001** ^2^
No	12 (15.2)	0 (0.0)	
Fall Risk (medium or high)			
Yes	66 (83.5)	44 (78.6)	0.464 ^1^
No	13 (16.5)	12 (21.4)	
Malnutrition/anorexia			
Yes	2 (2.5)	2 (3.6)	1.000 ^2^
No	77 (97.5)	54 (96.4)	
Obesity			
Yes	17 (21.5)	1 (1.8)	**0.001** ^1^
No	62 (78.5)	55 (98.2)	
Pressure ulcers at discharge			
Yes	16 (20.3)	9 (16.1)	0.538 ^1^
No	63 (79.7)	47 (83.9)	
History of recent fractures			
Yes	18 (22.8)	19 (33.9)	0.153 ^1^
No	61 (77.2)	37 (66.1)	

ADL, dependency in activities of daily life; CCI, Charlson Comorbidity Index; SD, Standard deviation; ^a^ Wald test; ^1^ Pearson Chi-Square test; ^2^ Fisher’s Exact Test; ^3^ Mann–Whitney test; ^4^ Student *t*-test. All significant variables are in bold.

**Table 2:** Most common chronic conditions and therapeutic subgroups prescribed according to age groups.

	Total (*n* = 135)	75–84 Years (*n* = 79)	≥85 Years (*n* = 56)	*p* ^1^
**Chronic conditions ^a^, *n* (%)**				
Hypertension	93 (68.9)	53 (67.1)	40 (71.4)	0.592
Constipation	46 (34.1)	28 (35.4)	18 (32.1)	0.690
Cerebrovascular disease	45 (33.3)	26 (32.9)	19 (33.9)	0.902
Dementia	44 (32.6)	25 (31.6)	19 (33.9)	0.780
Diabetes mellitus	44 (32.6)	32 (40.5)	12 (21.4)	**0.020**
Depression	42 (31.1)	30 (38.0)	12 (21.4)	**0.041**
Urinary incontinence	41 (30.4)	17 (21.5)	24 (42.9)	**0.008**
Congestive heart failure	34 (25.2)	18 (22.8)	16 (28.6)	0.445
Rheumatic disease	33 (24.4)	24 (30.4)	9 (16.1)	0.057
Arrhythmia	27 (20.0)	13 (16.5)	14 (25.0)	0.221
Benign prostatic hypertrophy	25 (56.8)	14 (56.0)	11 (57.9)	0.900
Renal disease	21 (15.6)	10 (12.7)	11 (19.6)	0.270
Any non-metastatic solid tumor	18 (13.3)	13 (16.5)	5 (8.9)	0.205
Chronic pulmonary disease	17 (12.6)	8 (10.1)	9 (16.1)	0.305
Hemiplegia	12 (8.9)	8 (10.1)	4 (7.1)	0.548
Recurrent urinary tract infection	12 (8.9)	6 (7.6)	6 (10.7)	0.530
**Most frequent drug prescribed (ATC_2 ^b^)**, ***n* (%)**				
Antithrombotic agents (B01)	96 (71.1)	61 (77.2)	35 (62.5)	0.063
Drugs for acid related disorders (A02)	93 (68.9)	54 (68.4)	39 (69.7)	0.873
Psycholeptics (N05)	90 (66.7)	52 (65.8)	38 (67.9)	0.805
Psychoanaleptics (N06)	74 (54.8)	51 (64.6)	23 (41.1)	**0.007**
Diuretics (C03)	71 (52.6)	40 (50.6)	31 (55.4)	0.588
Agents acting on the renin–angiotensin system (C09)	62 (45.9)	38 (48.1)	24 (42.9)	0.546
Lipid modifying agents (C10)	54 (40.0)	36 (45.6)	18 (32.1)	0.117
Drugs for constipation (A06)	45 (33.3)	27 (34.2)	18 (32.1)	0.805
Analgesics (N02)	43 (31.9)	26 (32.9)	17 (30.4)	0.754
Drugs used in diabetes (A10)	40 (29.6)	30 (38.0)	10 (17.9)	**0.012**
Beta-blocking agents (C07)	40 (29.6)	26 (32.9)	14 (25.0)	0.321
Cardiac therapy (C01)	34 (25.2)	19 (24.1)	15 (26.8)	0.718
Antiepileptics (N03)	30 (22.2)	25 (31.6)	5 (8.9)	**0.002**
Antianemic preparations (B03)	28 (20.7)	14 (17.7)	14 (25.0)	0.304

^a^ The chronic conditions reported by more than 5% of patients; ATC_2, ATC second level (therapeutic subgroup); ^b^ the therapeutic subgroups reported by more than 20% of patients. ^1^ Pearson Chi-Square. All significant variables are in bold.

**Table 3:** Prevalence of potentially inappropriate medications (PIMs) and potential prescribing omissions (PPOs) according to Screening Tool of Older People’s Prescriptions (STOPP) and Screening Tool to Alert to Right Treatment (START) criteria.

Criteria	75–84 Years (*n* = 79)	≥85 Years (*n* = 56)	*p*
**STOPP**			
Number of PIMs per patient			0.401 ^1^
Mean ± SD	2.9 ± 2.2	2.5 ± 1.8	
Median (P25; P75)	3 [1; 4]	2 [1; 3.75]	
0, *n* (%)	9 (11.4)	8 (14.3)	
1, *n* (%)	14 (17.7)	8 (14.3)	
2, *n* (%)	14 (17.7)	15 (26.8)	
3, *n* (%)	17 (21.5)	11 (19.6)	
≥4, *n* (%)	25 (31.6)	14 (25.0)	
Number of patients with ≥1 PIM, n (%)	70 (88.6)	48 (85.7)	0.618 ^2^
**START**			
Number of PPOs per patient			0.169 ^1^
Mean ± SD	1.8 ± 1.4	2.1 ± 1.4	
Median (P25; P75)	2 [1; 3]	2 [1; 3]	
0, *n* (%)	16 (20.3)	8 (14.3)	
1, *n* (%)	21 (26.6)	8 (14.3)	
2, *n* (%)	18 (22.8)	21 (37.5)	
3, *n* (%)	13 (16.5)	12 (21.4)	
≥4, *n* (%)	11 (13.9)	9 (12.5)	
Number of patients with ≥1 PPO, *n* (%)	63 (79.7)	48 (85.7)	0.372 ^2^
PIM Index			0.754 ^1^
Mean ± SD	0.312 ± 0.229	0.317 ± 0.215	
Median (P25; P75)	0.273 [0.143; 0.429]	0.333 [0.157; 0.441]	
STOPP-PIM or START-PPO, *n* (%)	76 (96.2)	54 (96.4)	1.000 ^3^
STOPP-PIM and START-PPO, *n* (%)	57 (72.2)	41 (75.0)	0.712 ^2^

SD, Standard deviation; ^1^ Mann–Whitney; ^2^ Pearson Chi-Square; ^3^ Fisher’s Exact Test.

**Table 4:** Most frequent potentially inappropriate medications (PIMs) and potential prescribing omissions (PPOs) (at least in 5% of the total population) and comparison between age groups.

		Total (*n* = 135)	75–84 Years (*n* = 79)	≥85 Years (*n* = 56)	*p*
**STOPP criteria**	Benzodiazepines as drugs that predictably increase the risk of falls	72 (53.3)	43 (54.4)	29 (51.8)	0.762 ^1^
	Benzodiazepines for ≥4 weeks	68 (50.4)	40 (50.6)	28 (50.0)	0.942 ^1^
	Neuroleptics as drugs that predictably increase the risk of falls	33 (24.4)	20 (25.3)	13 (23.2)	0.779 ^1^
	Tricyclic antidepressants with dementia, narrow angle glaucoma, cardiac conduction abnormalities, prostatism, or prior history of urinary retention	22 (16.3)	15 (19.0)	7 (12.5)	0.315 ^1^
	Anticholinergics/antimuscarinics in patients with delirium or dementia	20 (14.8)	13 (16.5)	7 (12.5)	0.524 ^1^
	Any drug prescribed without an evidence-based clinical indication	13 (9.6)	6 (7.6)	7 (12.5)	0.341 ^1^
	Any duplicate drug class prescription	11 (8.1)	7 (8.9)	4 (7.1)	1.000 ^2^
	Benzodiazepines with acute or chronic respiratory failure	11 (8.1)	6 (7.6)	5 (8.9)	0.762 ^2^
	Use of regular (as distinct from PRN) opioids without concomitant laxative	11 (8.1)	8 (10.1)	3 (5.4)	0.361 ^2^
	Proton pump inhibitors for uncomplicated peptic ulcer disease or erosive peptic oesophagitis at full therapeutic dosage for >8 weeks	10 (7.4)	4 (5.1)	6 (10.7)	0.318 ^2^
	Drugs likely to cause constipation in patients with chronic constipation where non-constipating alternatives are available	9 (6.7)	7 (8.9)	2 (3.6)	0.305 ^2^
	Hypnotic Z-drugs	9 (6.7)	5 (6.3)	4 (7.1)	1.000 ^2^
	Loop diuretic for treatment of hypertension with concurrent urinary incontinence	8 (5.9)	3 (3.8)	5 (8.9)	0.276 ^2^
	Initiation of tricyclic antidepressants as first-line antidepressant treatment	8 (5.9)	7 (8.9)	1 (1.8)	0.139 ^2^
	Neuroleptic antipsychotic in patients with behavioral and psychological symptoms of dementia unless symptoms are severe and other non-pharmacological treatments have failed	8 (5.9)	5 (6.3)	3 (5.4)	1.000 ^2^
	Long-acting opioids without short-acting opioids for break-through pain	7 (5.2)	5 (6.3)	2 (3.6)	0.699 ^2^
**START criteria**	Vitamin D supplement in older people who are housebound or experiencing falls or with osteopenia	59 (43.7)	28 (35.4)	31 (55.4)	0.022 ^1^
	Vitamin D and calcium supplement in patients with known osteoporosis and/or previous fragility fracture(s) and/or Bone Mineral Density T-scores more than −2.5 in multiple sites	38 (28.1)	19 (24.1)	19 (33.9)	0.209 ^1^
	Angiotensin converting enzyme inhibitor with systolic heart failure and/or documented coronary artery disease	26 (19.3)	14 (17.7)	12 (21.4)	0.590 ^1^
	Antiplatelet therapy with a documented history of coronary, cerebral or peripheral vascular disease	20 (14.8)	10 (12.7)	10 (17.9)	0.402 ^1^
	Appropriate beta-blocker with stable systolic heart failure	17 (12.6)	7 (8.9)	10 (17.9)	0.121 ^1^
	5-alpha reductase inhibitor with symptomatic prostatism, where prostatectomy is not considered necessary	16 (11.9)	10 (12.7)	6 (10.7)	0.731 ^1^
	Statin therapy with a documented history of coronary, cerebral or peripheral vascular disease, unless the patient’s status is end-of-life or age is >85 years	14 (10.4)	11 (13.9)	3 (5.4)	0.108 ^1^
	Alpha-1 receptor blocker with symptomatic prostatism, where prostatectomy is not considered necessary	11 (8.1)	4 (5.1)	7(12.5)	0.200 ^2^
	Laxatives in patients receiving opioids regularly	11 (8.1)	8 (10.1)	3 (5.4)	0.361 ^2^
	Non-tryciclic antidepressant drug in the presence of persistent major depressive symptoms	10 (7.4)	8 (10.1)	2 (3.6)	0.194 ^2^
	Regular inhaled β2 agonist or antimuscarinic bronchodilator for mild to moderate asthma or chronic obstructive pulmonary disease	8 (5.9)	4 (5.1)	4 (7.1)	0.718 ^2^

^1^ Pearson Chi-Square; ^2^ Fisher’s Exact Test.

**Table 5:** Predictors of potentially inappropriate medications (PIMs) and potential prescribing omissions (PPOs) according to Screening Tool of Older People’s Prescriptions (STOPP) and Screening Tool to Alert to Right Treatment (START) criteria, respectively, in study sample (*n* = 135) for the age groups 75–84 and ≥85 years old—univariate logistic regression.

	STOPP_PIM				START_PPO			
	75–84 Years Old Group (*n* = 79)		≥85 Years Old Group (*n* = 56)		75–84 Years Old Group (*n* = 79)		≥85 Years Old Group (*n* = 56)	
	OR (95% CI)	*p* ^a^	OR (95% CI)	*p* ^a^	OR (95% CI)	*p* ^a^	OR (95% CI)	*p* ^a^
**Demographic characteristics**								
Age (years)	-	-	-	-	-	-	-	**-**
Gender								
Male	0.54 (0.13; 2.20)	0.386	0.25 (0.05; 1.18)	0.079	9.23 (1.15; 74.42)	0.037	0.25 (0.05; 1.18)	0.079
Female	1		1		1		1	
**Medical history**								
Provenience/Origin								
Hospital	1.13 (0.28; 4.58)	0.863	3.15 (0.57; 17.48)	0.189	1.11 (0.37; 3.35)	0.857	0.47 (0.10; 2.23)	0.342
Residence	1		1		1		1	
Nursing home	-	-	-	-	-	-	-	-
Primary care	-	-	-	-	-	-	-	-
Other	-	-	-	-	-	-	-	-
Length of stay	1.00 (1.00; 1.01)	0.536	1.01 (0.99; 1.02)	0.293	1.00 (1.00; 1.01)	0.252	1.00 (0.99; 1.00)	0.183
Discharge to		0.454		0.962				0.943
Residence	-	-	-	-	-	-	3.50 (0.21; 58.77)	0.384
Death	-	-	-	-	-	-	3.50 (0.15; 84.69)	0.441
Another RNCCI response	-	-	-	-	-	-	2.25 (0.13; 38.81)	0.577
Social option/response	-	-	-	-	-	-	5.00 (0.21; 117.89)	0.318
Nursing home	-	-	-	-	-	-	3.00 (0.12; 73.64)	0.501
Other or not referred	-	-	-	-	-	-	1	
**Clinical features**								
Enteral nutrition	0.52 (0.09; 2.89)	0.451	-	-	2.83 (0.34; 23.91)	0.339	1.20 (0.13; 11.26)	0.876
Medication per patient	1.67 (1.19; 2.33)	0.003	1.56 (1.19; 2.24)	0.014	1.14 (0.94; 1.38)	0.172	1.23 (0.94; 1.60)	0.132
Number of doses	1.37 (1.08; 1.75)	0.011	1.40 (1.04; 1.88)	0.028	1.08 (0.93; 1.26)	0.309	1.19 (0.94; 1.50)	0.145
Comorbid diseases	1.05 (0.59; 1.87)	0.879	1.00 (0.51; 1.98)	1.000	1.68 (0.98; 2.87)	0.059	2.50 (1.02; 6.14)	0.046
CCI	1.18 (0.75; 1.86)	0.473	0.86 (0.53; 1.41)	0.554	1.34 (0.92; 1.96)	0.127	1.59 (0.85; 2.97	0.145
Geriatric syndromes								
Polypharmacy (≥5 drugs/day)	11.17 (1.84; 67.90)	0.009	9.00 (1.42; 57.12)	0.020	2.11 (0.35; 12.68)	0.416	3.67 (0.55; 24.51)	0.180
Comorbid diseases ≥ 2	0.95 (0.24; 3.84)	0.943	1.18 (0.26; 5.28)	0.827	2.37 (0.77; 7.34)	0.134	4.20 (0.77; 22.99)	0.098
CCI ≥ 6	1.32 (0.33; 5.35)	0.694	0.73 (0.13; 4.07)	0.723	2.75 (0.86; 8.84)	0.090	2.69 (0.59; 12.37)	0.203
Dependency in ADL	0.67 (0.08; 5.91)	0.719	-	-	3.64 (0.97; 13.57)	0.055	-	-
Fall risk (medium or high)	1.53 (0.28; 8.37)	0.622	1.27 (0.22; 7.26)	0.791	3.13 (0.86; 11.37)	0.084	1.27 (0.22; 7.26)	0.791
Malnutrition/anorexia	-	-	0.15 (0.01; 2.66)	0.196	0.24 (0.01; 4.09)	0.325	0.15 (0.01; 2.66)	0.196
Obesity	0.96 (0.18; 5.08)	0.957	-	-	0.78 (0.22; 2.82)	0.705	-	-
Pressure ulcers at discharge	2.18 (0.25; 18.84)	0.478	1.40 (0.15; 13.00)	0.767	4.69 (0.57; 38.50)	0.150	0.51 (0.09; 3.07)	0.464
History of recent fractures	1.04 (0.20; 5.50)	0.966	1.65 (0.30; 9.06)	0.567	-	-	4.20 (0.48; 36.98)	0.196
**Chronic conditions ^a^**								
Hypertension	1.02 (0.23; 4.46)	0.977	1.62 (0.34; 7.74)	0.548	0.91 (0.28; 2.96)	0.874	3.00 (0.65; 13.89)	0.160
Constipation	5.02 (0.60; 42.43)	0.138	1.50 (0.27; 8.29)	0.642	1.27 (0.39; 4.10)	0.695	3.84 (0.44; 33.86)	0.226
Cerebrovascular disease	0.20 (0.05; 0.88)	0.033	0.46 (0.10; 2.07)	0.308	1.10 (0.34; 3.58)	0.874	1.65 (0.30; 9.06)	0.567
Dementia	1.71 (0.33; 8.91)	0.522	4.20 (0.48; 36.98)	0.196	2.33 (0.60; 9.04)	0.223	4.20 (0.48; 36.98)	0.196
Diabetes mellitus	0.88 (0.21; 3.38)	0.798	2.08 (0.23; 18.80)	0.514	1.65 (0.51; 5.31)	0.401	2.08 (0.23; 18.80)	0.514
Depression	5.66 (0.67; 47.74)	0.111	2.08 (0.23; 18.80)	0.514	1.03 (0.33; 3.18)	0.965	-	-
Urinary incontinence	2.37 (0.28; 20.40)	0.432	6.44 (0.74; 56.43)	0.093	2.19 (0.45; 10.74)	0.335	2.54 (0.47; 13.87)	0.282
Congestive heart failure	1.04 (0.20; 5.50)	0.966	3.18 (0.36; 28.21)	0.299	2.38 (0.49; 11.65)	0.283	-	-
Rheumatic disease	0.86 (0.20; 3.76)	0.838	0.51 (0.09; 3.07)	0.464	0.67 (0.21; 2.11)	0.490	1.40 (0.15; 13.00)	0.767
Arrhythmia	-	-	0.50 (0.10; 2.41)	0.384	-	-	-	-
Benign prostatic hypertrophy	4.88 (0.43; 55.29)	0.201	0.25 (0.02; 2.84)	0.263	-	-	10.00 (0.84; 119.32)	0.069
Renal disease	-	-	1.84 (0.20; 16.76)	0.588	2.50 (0.29; 21.33)	0.402	0.69 (0.12; 4.01)	0.682
Any non-metastatic solid tumor	1.66 (0.19; 14.49)	0.649	-	-	0.82 (0.20; 3.40)	0.782	0.64 (0.06; 6.55)	0.704
Chronic obstructive pulmonary disease	-	-	0.12 (0.02; 0.62)	0.011	-	-	1.40 (0.15; 13.00)	0.767
Hemiplegia	0.89 (0.10; 8.19)	0.917	0.47 (0.04; 5.14)	0.534	1.88 (0.21; 16.45)	0.570	0.47 (0.04; 5.14)	0.534
Recurrent urinary tract infection	-	-	-	-	-	-	-	-

ADL, dependency in activities of daily life; CCI, Charlson Comorbidity Index; RNCCI, Portuguese National Network for Long-term Integrated Care; ^a^ Wald’s test; For the binary categorical variables, we considered the category “no” as a reference.

**Table 6:** Predictors of potentially inappropriate medications (PIMs) and potential prescribing omissions (PPOs) according to Screening Tool of Older People’s Prescriptions (STOPP) and Screening Tool to Alert to Right Treatment (START) criteria in study sample (*n* = 135) for the age groups 75–84 and ≥85 years old—multivariate logistic regression.

STOPP_PIM				
	75–84 Years Old Group (*n* = 79) ^1^		≥85 Years Old Group (*n* = 56) ^2^	
	Adjusted OR (95% CI)	*p* ^a^	Adjusted OR (95% CI)	*p* ^a^
Number of medications	1.71 (1.19–2.48)	0.004	1.44 (1.04–2.01)	0.028
Cerebrovascular disease	0.16 (0.03–0.91)	0.038	-	-
Chronic obstructive pulmonary disease	-	-	0.12 (0.02–0.90)	0.039
**START_PPO**				
	**75–84 Years Old Group (*n* = 79) ^3^**		**≥85 Years Old Group (*n* = 56) ^4^**	
	**Adjusted OR (95% CI)**	***p* ^a^**	**Adjusted OR (95% CI)**	***p* ^a^**
Gender				
Male	14.41 (1.55; 134.47)	0.019	-	-
Female	1			
Fall risk (medium or high)	5.72 (1.21; 27.05)	0.028	-	-
Comorbid diseases	-	-	2.50 (1.02; 6.14)	0.046

CI, confidence interval; OR, odd ratio; ^a^ Wald’s test; For the binary categorical variables, we considered the category “no” as a reference; The ORs were adjusted for sex and for the significant variables in Table 5 and we used a forward variable selection method based on the likelihood ratio (significance level at 5% for a variable entering and 10% for its removal). ^1^ Cox&Snell R^2^: 0.197; Nagelkerke R^2^: 0.388; Hosmer and Lemeshow: *p* = 0.205; Area under ROC curve: 0.840; Sensitivity = 81.4% and Specificity = 77.8% for the cut-off probability 0.861. ^2^ Cox&Snell R^2^: 0.208; Nagelkerke R^2^: 0.372; Hosmer and Lemeshow: *p* = 0.254; Area under ROC curve: 0.789; Sensitivity = 66.7% and Specificity = 75.0% for the cut-off probability 0.884. ^3^ Cox&Snell R^2^: 0.145; Nagelkerke R^2^: 0.229; Hosmer and Lemeshow: *p* = 0.689; Area under ROC curve: 0.724; Sensitivity = 38.1%; Specificity = 93.7% for the cut-off probability 0.835 ^4^ Cox&Snell R^2^: 0.085; Nagelkerke R^2^: 0.152; Hosmer and Lemeshow: *p* = 0.942; Area under ROC curve: 0.724; Sensitivity = 58.3% and Specificity = 75.00% for the cut-off probability 0.872.

## Data Availability

The original contributions presented in this study are included in the article. Further inquiries can be directed to the corresponding authors.
